# Definition of a Novel Plasmid-Based Gene Transfection Protocol of Mammalian Skeletal Muscles by Means of In Vivo Electroporation

**DOI:** 10.3390/ijms21186494

**Published:** 2020-09-05

**Authors:** Enrico P. Spugnini, Manuel Scimeca, Bruno Amadio, Giancarlo Cortese, Maurizio Fanciulli, Bruno Vincenzi, Antonio De Luca, Alfonso Baldi

**Affiliations:** 1 Biopulse srl, 80100 Naples, Italy; spugnini.vet@gmail.com; 2 Orchidealab srl, 00100 Rome, Italy; 3 Department of Biomedicine and Prevention, University of Rome “Tor Vergata” 00100 Rome, Italy; manuel.scimeca@uniroma2.it; 4 UOSD SAFU Department of Research, Diagnosis and Innovative Technologies, Translational Research Area, IRCCS Regina Elena National Cancer Institute, 00100 Rome, Italy; Bruno.amadio@ifo.gov.it (B.A.); Giancarlo.cortese@ifo.gov.it (G.C.); maurizio.fanciulli@ifo.gov.it (M.F.); 5 Department of Medical Oncology, Campus Bio-Medico University, 00100 Rome, Italy; B.Vincenzi@unicampus.it; 6 Department of Mental and Physical Health and Preventive Medicine, Section of Human Anatomy, University of Campania “L. Vanvitelli”, 80100 Naples, Italy; antonio.deluca@unicampania.it; 7 Department of Environmental, Biological, Pharmaceutical Sciences and Technologies, University of Campania “L. Vanvitelli”, 81100 Caserta, Italy

**Keywords:** biphasic pulses, DNA plasmid, Green Fluorescent Protein (GFP), electroporation

## Abstract

We describe an original electroporation protocol for in vivo plasmid DNA transfection. The right hind limbs of C57 mice are exposed to a specifically designed train of permeabilizing electric pulses by transcutaneous application of tailored needle electrodes, immediately after the injection of pEGFP-C1 plasmid encoding GFP (Green Fluorescente Protein). The electroporated rodents show a greater GFP expression than the controls at three different time points (4, 10, and 15 days). The electroporated muscles display only mild interstitial myositis, with a significant increase in inflammatory cell infiltrates. Finally, mild gait abnormalities are registered in electroporated mice only in the first 48 h after the treatment. This protocol has proven to be highly efficient in terms of expression levels of the construct, is easy to apply since it does not require surgical exposure of the muscle and is well tolerated by the animals because it does not cause evident morphological and functional damage to the electroporated muscle.

## 1. Introduction

The observation that muscle cells can take up and express foreign DNA for prolonged periods in vivo has been well documented for more than 30 years [1]. This has generated the idea that the methodology could be applied for vaccination and gene therapy. The gene transfer methods for in vivo ectopic expression are essentially classified into viral or non-viral [2,3]. The use of viral vectors, such as genetically modified adenoviruses, lentiviruses, adeno-associated viruses, and retroviruses has the great advantage of guaranteeing high transfection efficiency and stability [4]. It must be considered, however, that viral methods show various contraindications, including high costs, cytotoxicity, the possibility of causing mutagenesis, and the limitation in the size of the transgene to be inserted [5]. Quite the reverse, non-viral techniques are significantly less expensive, give the possibility to insert larger transgenes, display lower cytotoxicity and immunogenic responses, and safely limit the likelihood of insertional mutagenesis [3]. Nevertheless, it is well documented that plasmid-based DNA transfection by direct injection into skeletal muscle has a very low efficiency [6,7], and this narrows its experimental and therapeutic use for in vivo application.

Several factors have been called into question to justify the low transfection efficiency of plasmid DNA through direct injection. Among these, we remember the structure of plasmid constructs, the parameters used for the injection included, the volume and type of vehicle, and the injection speed [8]. Consistently, numerous experimental strategies have been used to improve transfection efficacy. Among others, we mention the use of solutions capable of inhibiting nuclease-mediated digestion of DNA, the variation of the inoculation parameters and, finally yet importantly, the application of an electric field on the injection site [9,10,11,12,13].

An important turning point in this issue has been, indeed, the demonstration that the application of an electric field is able to significantly improve the expression of transgene in skeletal muscle cells in vivo [8]. Nonetheless, several different electroporation protocols have been proposed to date, but there are numerous variables that have been seen that can alter the transfection efficiency [14]. Among the others, we can indicate the voltage applied, the duration of electric pulses, the number of electric pulses, pre-treatment of the muscle, and the DNA solute used for injection. Finally, it is necessary to evaluate the damage caused by electroporation to the treated muscles, which can alter the efficiency and repeatability of transfection [5].

Our research group has acquired great experience in the methods of delivering chemotherapy drugs in vivo to tumor tissues through electrical impulses (electrochemotherapy) both in animal models and in humans [15,16,17,18]. Here, we illustrate an original protocol of plasmid-based DNA electroporation in skeletal muscle tissue in vivo using an innovative electrical impulse scheme. This protocol not only proves to be very efficient but also is easy to apply and does not cause significant damage to the electroporated muscle fibers.

## 2. Results

**Figure 1:**
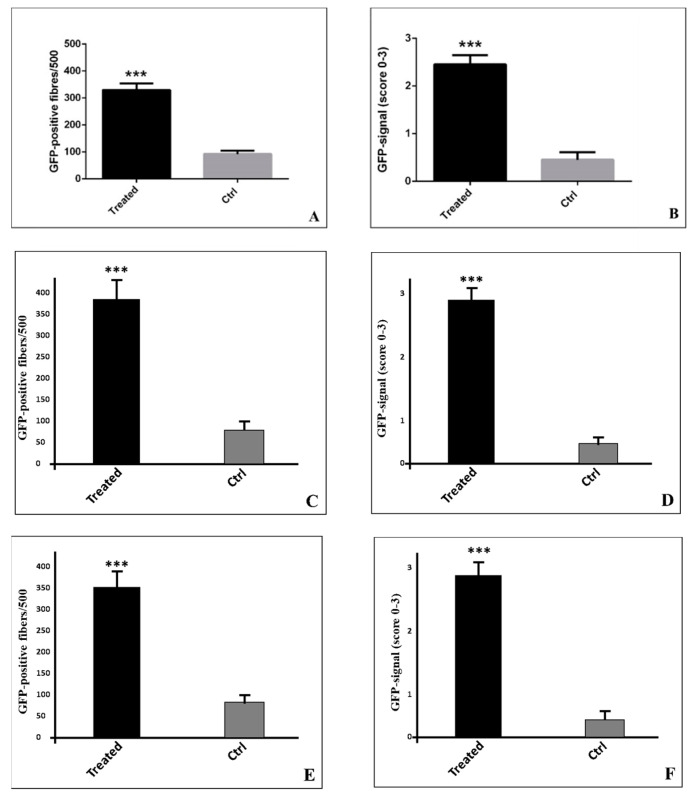
Evaluation of the GFP (Green Fluorescent Protein) signal in skeletal muscle fibers. (**A**) Graph shows the number of GFP-positive muscle fibers in the treated and control groups (α = 0.05) at day 4. (**B**) Graph displays the GFP signal intensity in the treated and control groups (α = 0.05) at day 4. (**C**) Graph shows the number of GFP-positive muscle fibers in the treated and control groups (α = 0.05) at day 10. (**D**) Graph displays the GFP signal intensity in the treated and control groups (α = 0.05) at day 10. (**E**) Graph shows the number of GFP-positive muscle fibers in the treated and control groups (α = 0.05) at day 15. (**F**) Graph displays the GFP signal intensity in the treated and control groups (α = 0.05) at day 15. Asterisks indicate a significant difference between values.

Animals were randomly allocated to one of the two groups: (a) the treated group receiving electroporation in the right limb, sacrificed 4, 10, and 15 days after the treatment; (b) the control group not receiving electroporation in the right limb, sacrificed 4, 10, and 15 days after the treatment. Immunofluorescence analysis was performed to evaluate the number of skeletal fibers showing a positivity for GFP (Green Fluorescent Protein) (number of GFP-positive fibers on 500). Additionally, we evaluated the distribution of the GFP signal (homogeneous versus focal) and the intensity of the signal by assigning a score from 0–3 according to the signal intensity, as described in the Methods Section. A significant increase in the number of GFP positive muscle fibers in the treated group (329.6 ± 80.59 at day 4, 365.96 ± 91.19 at day 10, and 353,6 ± 89.49 at day 15) compared to the control (92.36 ± 42.10 at day 4, 81.45 ± 38.40 at day 10, and 85.45 ± 35.50 at day 15) (*p <* 0.0001) was observed. Agreeing with the number of GFP-positive fibers, a significant increase in the GFP signal (score 0–3) was observed in the treated group (2.45 ± 0.65 at day 4, 2.35 ± 0.55 at day 10, and 2.255 ± 0.55 at day 15) when compared with the control (0.45 ± 0.52 at day 4, 0.40 ± 0.32 at day 10, and 0.45 ± 0.52 at day 15) (*p <* 0.0001). Noteworthy in the treated group, positive fibers showed a very homogeneous signal. Figure 1 uses the histograms to show the various scores in the different time points of the experiment.

**Figure 2:**
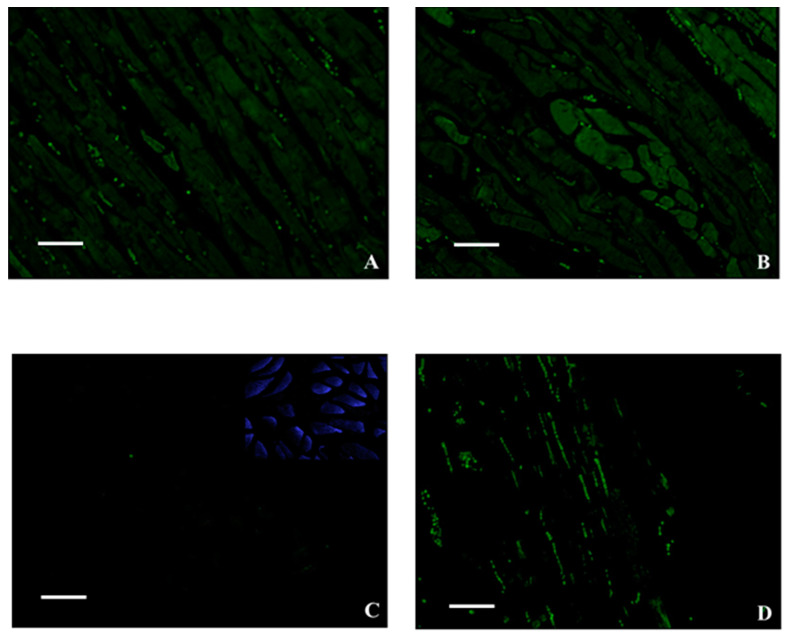
Representative fluorescence staining for GFP. (**A**) Image shows numerous GFP-positive fibers in a treated mouse’s hind right leg. (**B**) High GFP-signal in an electroporated skeletal muscle of a mouse’s hind right leg. (**C**) Image displays no/rare GFP-positive fibers in a control mouse’s hind right leg. Square shows DAPI-signal. (**D**) Very low GFP-signal in a control mouse’s hind right leg; note a background signal (erythrocytes) in a negative region. Scale bar represents 100 µm for all images.

Figure 2 shows representative fluorescence staining for GFP in treated and control animals.

**Figure 3:**
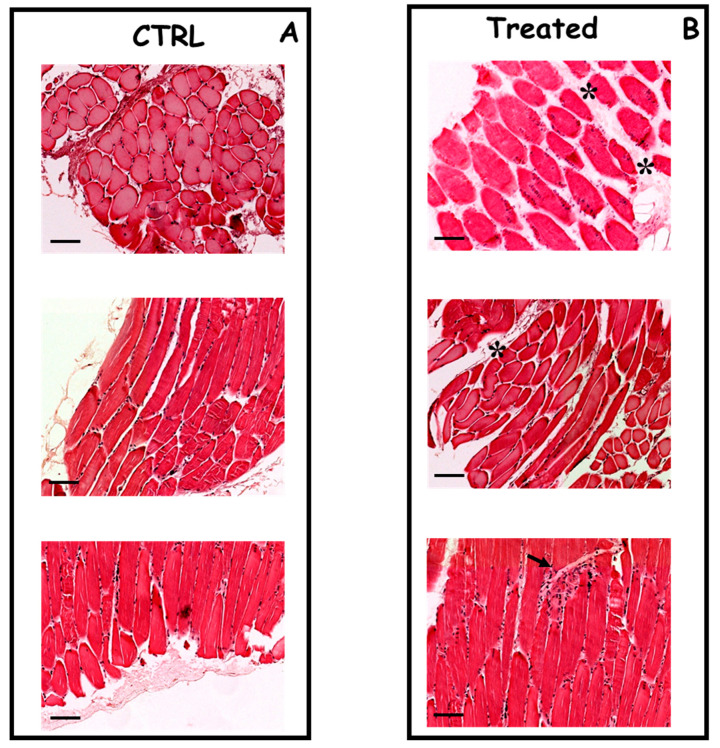
Histopathological analysis of skeletal muscle of mice untreated or treated by needle electrodes. Panel (**A**) cross-sections of skeletal muscle from an untreated mouse, showing no significant histopathological alterations (hematoxylin and eosin, original magnification ×20); Panel (**B**) cross-sections of skeletal muscle from a mouse treated by caliper electrodes showing a focus of mild mononuclear inflammatory infiltrate (arrow) and a slight deviation of the fibers (asterisks). (hematoxylin and eosin, original magnification ×20). Scale bar represents 100 μm for all images.

Histological analysis of the muscles was performed analyzing hematoxylin and eosin staining as well as Masson’s trichrome staining. Indeed, the needle arrays used for the electroporation caused only mild interstitial myositis and a slight deviation of the fibers, with no necrosis, neither phagocytosis of the muscle fibers, nor fibrosis (Figure 3).

**Figure 4:**
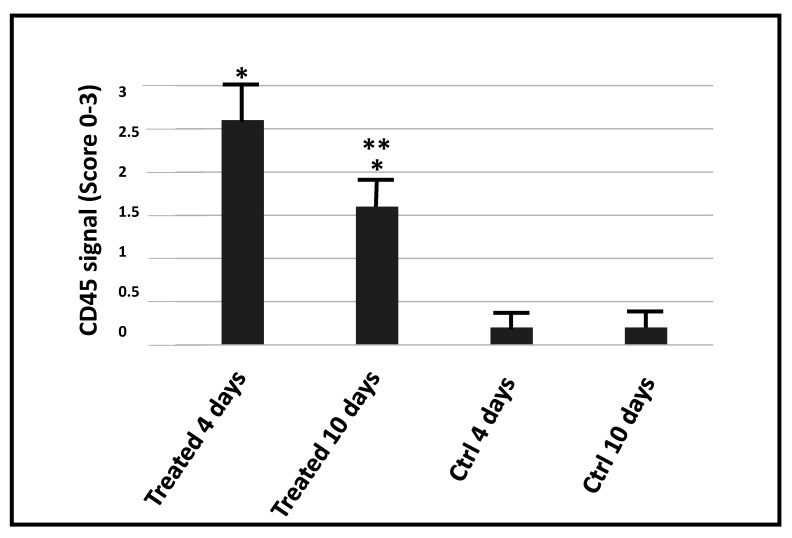
Evaluation of CD45 immunohistochemical staining in skeletal muscle. The graph shows the score of CD45 immunoreactivity in muscles treated with electroporation and in control animals (Ctrl). The single asterisk indicates a statistically significant difference in the scores between treated and untreated mice at the two time points, while a double asterisk indicates a statistically significant difference in the scores between the two treated groups.

Immunohistochemistry for CD45 was used to analyze the inflammatory response of skeletal tissue undergoing electroporation. Indeed, a significant increased level of CD45 expression was observed in treated skeletal tissue in respect to the control at both 4 and 10 days after electroporation (*p* = 0.006 and *p* = 0.0011 respectively). Interestingly, the level of CD45 expression significantly decreased in the treated animals at 10 days after electroporation in respect to the animal treated at 4 days after electroporation (*p* = 0.003) (Figure 4).

Mice were clinically evaluated for electroporation-induced side effects, including skin burns, ulcerations, swelling and gait abnormalities. Cutaneous side effects were not observed by the investigators. However, gait abnormalities were observed and scored according to Table 1. Fully, mice in group 1 (injection alone) showed a grade 3 score for 24 h; mice in the electroporation cohort exhibited grade 3 and grade 2 side effects that lasted for 48 h. Finally, no mice displayed a grade 1 or grade 0 score. All rodents successfully recovered from treatment side effects within 48 h.

All the results point out that the pulse generator and the insertional probe were able to maintain stable pulses among the experimental mice, and that the delivery within the muscles was constant and consistent among all the laboratory animals.

## 3. Discussion

Gene delivery, a process of introducing foreign functional nucleic acids into target cells, has proven to be a very promising tool for inducing specific gene expression in host cells. Efficient yet non-invasive delivery of biomolecules in a high-throughput manner has long fascinated the scientific community; however, intracellular delivery of foreign molecules including nucleic acids and proteins, remains challenging. Electroporation has been adopted in gene delivery for decades, and is currently widely used for transfection of different types of cells. DNA vaccine technology is based on the fact that it is capable of inducing an immune response to recombinant antigens encoded by plasmid DNA and expressed in vivo. Actually, the host’s cellular machinery complex is capable of expressing the genes encoded by the plasmid, and of promoting the generation of antigens that can be processed and presented to the molecules of the two major histocompatibility complexes [20]. Consequently, these antigens are effectively recognized by the immune system and can induce complete and effective immunization [21]. Despite the great potential, to date very few plasmid DNA vaccines are registered and are only for use in the veterinary field [2]. The reasons for this must be sought in various critical points of this technology, which make its use in clinical practice still difficult, despite the enormous potential. Comprehensively, one of the most important critical points is represented by the low immunogenicity of DNA vaccines in humans, possibly caused by the trouble to upscale the DNA vaccine quantities used in small animal systems [20]. Therefore, one of the major goals to achieve is to optimize the efficiency of transfection.

Currently, the use of in vivo electroporation is a well-established delivery method for targeting skeletal muscle [22,23]. Here, we describe an original protocol to efficiently transfect plasmid DNA in mammalian skeletal muscles using in vivo electroporation. The GFP signal was used to assess the level of transfection. Indeed, the expression of GFP was significantly higher in electroporated muscles in respect to the control at 4, 10, and 15 days after transfection. An important point to be addressed is to determine damages caused by electroporation to the treated muscles, which can alter the efficiency and repeatability of transfection [5]. Using histological analysis, we demonstrated that the needle arrays used for the electroporation caused only mild interstitial myositis, with no necrosis, neither phagocytosis of the muscle fibers, nor fibrosis. Moreover, we performed immunohistochemical analysis with CD45 to better visualize the recruitment of inflammatory cells in the treated muscles. Electroporation was able to elicit an inflammatory response in the area close to the application of the needle arrays. However, the recruitment of inflammatory cells significantly decreased after 10 days of treatment, while remaining clearly above the levels found in non-electroporated muscles. Regarding side effects, we observed only mild gait abnormalities that lasted only for the first 48 h after the treatment.

It is well known that cytoplasmic trafficking and passage through the nuclear membrane are the main switches to attain an effective DNA transfection both in vitro and in vivo [3]. This translocation across the cytoplasm denotes a substantial block to gene delivery. Indeed, cytoplasmic nucleases are able to degrade free DNA; moreover, significant numbers of proteins can be found associated with the transfected plasmid [24]. The high expression of GFP by muscle cells maintained after 10 days from electroporation in our experiment suggests efficient DNA transfer within the nucleus and expression.

Concerning the problem of prolonged expression, it must be underlined that one of the advantages of adeno-associated/retroviral gene delivery, a “competitor” of the technique presented here, is indeed the potential for long term expression [3,4]. During our experimental setting, we have investigated expression of the plasmid DNA only at 4 and 10 days after electroporation. It would be necessary to examine how long expression can be maintained in future experimentations. In addition, as expression is likely to be temporary, it would be worthwhile to examine the effects of repeated transfection/electroporation since it is important to achieve sustained transgene expression. Moreover, it would be important to compare the efficacy of the gene expression protocol described in this article to viral methods, as well as non-viral methods that make use of common non-viral vectors (e.g., polyethylenimine, lipofectamine, DOTAP/Cholesterol, etc.). Along these lines, we will set up new experimentations to see if this electroporation technique can be combined with either viral or non-viral methods to yield an even more efficient transfection.

Finally, it should be underlined that our protocol has the great advantage of being easily applied, as it does not require surgical exposure of the muscle tissue to be electroporated, rather only the transcutaneous application of needle electrodes designed specifically for this function. The single use replaceable needle array terminal ensures the repeatability of the electroporation parameters changing the needles that might bend or become oxidized during the experimental procedures. Moreover, this will allow for the adoption of single use arrays when novel electroporation protocols will translate into clinics. This makes the protocol more easily adaptable to potential clinical applications. The plasmid DNA transfection protocol by electroporation, described in this article, proves highly efficient in terms of expression levels of the construct. Furthermore, by not requiring surgical exposure of the muscle to be electroporated, it proves easy to apply in potential clinical applications. Finally, this protocol does not cause evident histological damage to the electroporated muscle, while eliciting an inflammatory response of the tissue, which is believed to be important for amplifying the immunogenic potential of the electroporated DNA [20].

## 4. Materials and Methods

### 4.1. Plasmid DNA Preparation

The pEGFP-C1 plasmid (Takara Bio Europe, Saint-Germain-en-Laye, France) encoding GFP (Green Fluorescent Protein) was adopted to assess transfection efficacy [25]. Plasmid DNA was amplified using standard protocols to yield a concentration of 5 μg/μL.

### 4.2. Intra-Muscular Injection

Female C57 mice (8–10 weeks old; weight 18–25 g) were obtained from Charles Rivers Laboratories (Milan, Italy). Mice were housed in individual cages, at a temperature of 22 °C and a photoperiod of 12 h of light and 12 h of darkness for two weeks before each experiment. Animals had ad libitum water and food. Each experiment used 10 mice per treatment arm and was repeated twice for confirmation of the obtained data. All procedures were in accordance with institutional guidelines under the control of the Italian Ministry of Public Health (Italian Law D.lgs 26/2014) and conform to Directive 2010/63/EU and with the Guide for the Care and Use of Laboratory Animals. The animal study was approved by the Italian Ministry of Health (approval number 246-2020 PR; date of approval 14 April 2020). Mice were anesthetized with Zoletil 50 (1:1 tiletamine/zolazepam mixture, Virbac, Milano, Italy) in combination with Xilor (20 mg/mL xylazine, BIO 98 Srl, Milan, Italy) as described elsewhere [26]. The Zoletil 50 powder was reconstructed by 5 mL of the applied solvent, and the stock solution was mixed with Xilor and water for injection [24]. The anesthetic mixture was administered intramuscularly (0.05 mL/mouse) at a dose level of approximately 25 mg/kg (both for tiletamine and for zolazepam), and 7.5 mg/kg (for xylazine). The loss of the righting reflex, the pedal reflex, and the response to tail pinch was considered the onset of anesthesia, occurring within 6–8 min. The right hind limb was shaved, and then 10 µL of a 2 mg/mL hyaluronidase solution was injected using a 1″ long 33-gauge sterile needle. After 5 min, 50 µg of plasmid DNA in a final volume of 10 μL was injected at the same site. Then, animals were randomly allocated to one of the two groups: (a) the treated group receiving electroporation, sacrificed 4, 10, and 15 days after the treatment; (b) the control group not receiving electroporation, sacrificed 4, 10, and 15 days after the treatment.

### 4.3. Electrical and Instrumental Parameters for Electroporation

**Figure 5:**
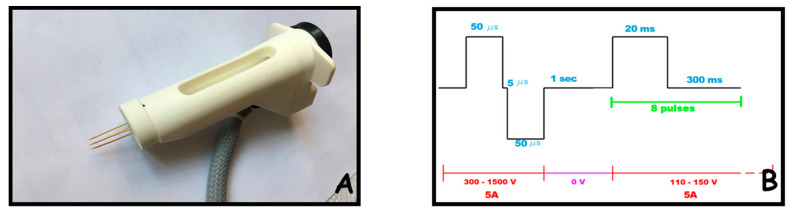
The original needle electrodes and electrical parameters designed for electroporation. Panel (**A**) the needle array electrode used for electroporation is depicted; it consists of a handle where a syringe can be lodged surrounded by a changeable four-needle array, connected to the impulse generator. Panel (**B**) schematic representation of the electrical parameters used for electroporation.

The trains of electric pulses were administered with a clinical electroporator certified for veterinary use (Onkodisruptor^®^, Biopulse srl., Italy). The first impulse was applied immediately after the plasmid injection. All told, 9 impulses were applied. Each train of impulses was made up of an initial biphasic pulse with a total duration of 100 μs (with a neutral interpulse of 5 μs), a 1 s pause, then followed by 8 monophasic pulses of 20 ms separated by neutral interpulses of 300 ms. The maximum current intensity was set at 5A, while the fixed voltage was set at 1200 V for the first biphasic wave, and at 110 V for the train of 8 monophasic pulses. Figure 5A depicts the needle array electrode used for electroporation, which consists of a single use needle array that can be changed after every treatment. This configuration is already set for clinical translation. Figure 5B reports the schematic representation of the train of impulses adopted for electroporation.

### 4.4. Evaluation of Electroporation-Associated Side Effects

Mice were clinically evaluated for electroporation-induced side effects, including skin burns, ulcerations, swelling and gait abnormalities. All evaluations were independently performed by two authors (EPS and AB). Fully, concerning gait abnormalities, the score depicted in Table 1 was used [19].

### 4.5. Histology, Immunohistochemistry and GFP Analysis by Fluorescence

Following sacrifice, small fragments of skeletal muscle (1.0 × 1.0 × 0.4 cm) from the right hind limbs were taken in the subcutaneous region of each mouse. Samples were fixed overnight in 4% paraformaldehyde at 4 °C. Then, each fragment was washed in PBS, cryoprotected in 30% sucrose and snap-frozen in an optimal cutting temperature (OCT) solution. Cryosections (8 µm) were placed on a histological slide and nuclear countered with DAPI [27]. Each slide was observed using a fluorescence microscope (Eclipse e1000–Nikon) to evaluate the number of skeletal fibers showing a positivity for GFP. Fully, the GFP signal was evaluated by analyzing 10 areas at 40× magnification. Specifically, a GFP positive signal in at least 5 neighboring regions or 8 regions regardless of distance was considered as homogeneous. Samples with a GFP positive signal in greater than or equal to one area were considered negative. Samples that do not meet the above-mentioned conditions were considered as a focal positivity for the GFP signal. Regarding histological analysis, cryosections (8 µm) were placed on histological slides and colored with Hematoxylin and Eosin and with Masson’s Trichrome staining, following standard procedures. Immunohistochemistry was performed as previously described [28,29,30]. Briefly, tissue sections were processed with the streptavidin-biotin-immunoperoxidase method (Universal kit, DAKO, Carpinteria, CA, USA). Unmasking was performed at 95 °C with EDTA pH 7.8 for 20 min. Rabbit anti-human CD45 antibody (ab10558 form Abcam, Cambridge, UK) was used at a 1:200 dilution and incubated 1 h rt. Diaminobenzidine was used as the final chromogen and hematoxylin as the nuclear counterstain. Two observers (M.S. and A.B.), blinded to treatment conditions, evaluated the staining pattern of the proteins separately and quantitated the protein expression in each specimen by scanning the entire section and estimating the number of positive cells at the high-power field 10 × 20.

### 4.6. Statistical Analysis

Statistical analysis and graphs for the interpretation of the data about GFP expression were performed using GraphPad Prism 5 Software (www.graphpad.com). The t test (Mann–Whitney) was used for evaluating the difference among the GFP-positive fibers and the GFP-intensity of the signal between the treated and control groups (α = 0.05). SPSS software (www.spss.it) (version 17.00, SPSS, Chicago, IL, USA) was used for statistical analysis of the data about CD45 expression. Descriptive analysis was performed using median values and a 95% Confidence Interval (CI) and percentages when appropriate. Differences between the two groups of animals were assessed using the Mann–Whitney U test for non-parametric continuous variables. Moreover, the different distribution of other variables between groups was assessed using a chi-square test. A *p* value of less than 0.05 was considered to indicate statistical significance.

## 5. Conclusions

The novelties of this article, in respect to what is well established in the scientific literature concerning in vivo electroporation delivery methods [22,23] were the following: (a) the definition of original electrical parameters; (b) the creation of tailored replaceable needle arrays; (c) the fact that this method does not require surgical exposure of the muscle. The final result is the definition of an electroporation delivery method highly efficient in terms of expression levels of the construct, is easy to apply and well tolerated by the animals because it does not cause evident morphological and functional damage to the electroporated muscle. Further experiments are ongoing in our laboratory to better define the experimental conditions suitable for an even more efficient transfection, and to propose the clinical use of this protocol.

## Figures and Tables

**Table 1:** Gait scores for mice (adapted from ref. [19]).

Gait Score	Description
4	Normal
3	Minimal impairment
2	Moderate impairment
1	Significant impairment
0	Unable to walk on the examined limb

## Abbreviation

GFP: Green Fluorescent Protein

## References

[1] Wolff J.A., Malone R.W., Williams P., Chong W., Acsadi G., Jani A., Felgner P.L. (1990). Direct gene transfer into mouse muscle in vivo. Science.

[2] Flingai S., Czerwonko M., Goodman J., Kudchodkar S.B., Muthumani K., Weiner D.B. (2013). Synthetic DNA vaccines: Improved vaccine potency by electroporation and co-delivered genetic adjuvants. Front. Immunol..

[3] Wells D.J. (2006). Viral and non-viral methods for gene transfer into skeletal muscle. Curr. Opin. Drug Discov. Develop..

[4] Gregoveric P., Biankinship M.J., Chamberlain J.S. (2004). Viral vectors for gene transfer to striated muscle. Curr. Opin. Mol. Ther..

[5] Schertzer J.D., Plant D.R., Lynch G.S. (2005). Optimizing plasmid-based gene transfer for investigating skeletal muscle structure and function. Mol. Ther..

[6] McMahon J.M., Wells D.J. (2004). Electroporation for gene transfer to skeletal muscles: Current status. BioDrugs.

[7] Peri D., Deville M., Poignard C., Signori E., Natalini R. (2020). Numerical optimization of plasmid DNA delivery combined with hyaluronidase injection for electroporation protocol. Comput. Methods Programs Biomed..

[8] Schertzer J.D., Lynch G.S. (2008). Plasmid-based gene transfer in mouse skeletal muscle by electroporation. Methods Mol. Biol..

[9] Andre F.M., Cournil-Henrionnet C., Vernerey D., Opolon P., Mir L.M. (2006). Variability of naked DNA expression after direct local injection: The influence of the injection speed. Gene Ther..

[10] Hartikka J., Bozoukova V., Jones D., Mahajan R., Wloch M.K., Sawdey M., Buchner C., Sukhu L., Barnhart K.M., Abai A.M. (2000). Sodium phosphate enhances plasmid DNA expression in vivo. Gene Ther..

[11] Lavigne M.D., Yates L., Coxhead P., Górecki D.C. (2008). Nuclear-targeted chimeric vector enhancing nonviral gene transfer into skeletal muscle of Fabry mice in vivo. FASEB J..

[12] Molnar M.J., Gilbert R., Lu Y., Liu A.B., Guo A., Larochelle N., Orlopp K., Lochmuller H., Petrof B.J., Nalbantoglu J. (2004). Factors influencing the efficacy, longevity, and safety of electroporation-assisted plasmid-based gene transfer into mouse muscles. Mol. Ther..

[13] Niedzinski E.J., Chen Y.J., Olson D.C., Parker E.A., Park H., Udove J.A., Scollay R., McMahon B.M., Bennett M.J. (2003). Enhanced systemic transgene expression after nonviral salivary gland transfection using a novel endonuclease inhibitor/DNA formulation. Gene Ther..

[14] DiFranco M., Quinonez M., Capote J., Vergara J. (2009). DNA transfection of mammalian skeletal muscles using in vivo electroporation. J. Vis. Exp..

[15] Pasquali P., Freites-Martinez A., Gonzalez S., Spugnini E.P., Baldi A. (2017). Successful treatment of plantar warts with intralesional bleomycin and electroporation: Pilot prospective study. Dermatol. Pract. Concept.

[16] Pasquali P., Spugnini E.P., Baldi A. (2018). Successful Treatment of a Keratoacanthoma with Electrochemotherapy: A Case Report. Dermatol. Ther..

[17] Spugnini E.P., Fais S., Azzarito T., Baldi A. (2017). Novel Instruments for the Implementation of Electrochemotherapy Protocols: From Bench Side to Veterinary Clinic. J. Cell Physiol..

[18] Spugnini E.P., Baldi A. (2019). Electrochemotherapy in Veterinary Oncology: State-of-the-Art and Perspectives. Vet. Clin. North Am. Small Animal Pract..

[19] Lakes E.H., Allen K.D. (2016). Gait analysis methods for rodent models of arthritic disorders: Reviews and recommendations. Osteoarthr. Cartil..

[20] Hobernik D., Bros M. (2018). DNA Vaccines—How Far From Clinical Use?. Int. J. Mol. Sci..

[21] Grunwald T., Ulbert S. (2015). Improvement of DNA vaccination by adjuvants and sophisticated delivery devices: Vaccine-platforms for the battle against infectious diseases. Clin. Exp. Vaccine Res..

[22] Roche J.A., Ford-Speelman D.L., Ru L.W., Densmore A.L., Roche R., Reed P.W., Bloch R.J. (2011). Physiological and histological changes in skeletal muscle following in vivo gene transfer by electroporation. Am. J. Physiol. Cell Physiol..

[23] Sokolowska E., Blachnio-Zabielska A.U. (2019). A critical review of electroporation as a plasmid delivery system in mouse skeletal muscle. Int. J. Mol. Sci..

[24] Bai H., Lester G.M., Petishnok L.C., Dean D.A. (2017). Cytoplasmic transport and nuclear import of plasmid DNA. Biosci. Rep..

[25] Esposito V., Manente L., Lucariello A., Perna A., Viglietti R., Gargiulo M., Parrella R., Parrella G., Baldi A., De Luca A. (2012). Role of FAP48 in HIV-associated lipodystrophy. J. Cell Biochem..

[26] Khokhlova O.N., Tukhovskaya E.A., Kravchenko I.N., Sadovnikova E.S., Pakhomova I.A., Kalabina E.A., Lobanov A.V., Shaykhutdinova E.R., Ismailova A.M., Murashev A.N. (2017). Using Tiletamine-Zolazepam-Xylazine Anesthesia Compared to CO_2_-inhalation for Terminal Clinical Chemistry, Hematology, and Coagulation Analysis in Mice. J. Pharmacol. Toxicol. Methods.

[27] Esposito T., Lucariello A., Hay E., Contieri M., Tammaro P., Varriale B., Guerra G., De Luca A., Perna A. (2019). Effects of curcumin and its adjuvant on TPC1 thyroid cell line. Chem. Biol. Interact..

[28] Lucariello A., Trabucco E., Boccia O., Perna A., Sellitto C., Castaldi M.A., De Falco M., De Luca A., Cobellis L. (2015). Small leucine rich proteoglycans are differently distributed in normal and pathological endometrium. In Vivo.

[29] De Luca A., De Falco M., Fedele V., Cobellis L., Mastrogiacomo A., Laforgia V., Tuduce I.L., Campioni M., Giraldi D., Paggi M.G. (2004). The serine protease HtrA1 is upregulated in the human placenta during pregnancy. J. Histochem. Cytochem..

[30] Baldi A., Lombardi D., Russo P., Palescandolo E., De Luca A., Santini D., Baldi F., Rossiello L., Dell’Anna M.L., Mastrofrancesco A. (2005). Ferritin contributes to melanoma progression by modulating cell growth and sensitivity to oxidative stress. Clin. Cancer Res..

